# Effects of the Addition of Starches and Storage Duration on the Chemical Composition, Physicochemical, Rheological, and Sensory Properties of Yoghurt: A Review

**DOI:** 10.1155/ijfo/4984917

**Published:** 2026-06-23

**Authors:** Ngoualem Kégah Franklin, Ndjouenkeu Robert

**Affiliations:** ^1^ Department of Food Science and Technology, Faculty of Agriculture and Veterinary Medicine, University of Buea, Buea, Southwest Region, Cameroon, ubuea.cm; ^2^ Department of Food Science and Nutrition, National School of Agro-Industrial Sciences, University of Ngaoundéré, Ngaoundéré, Adamawa, Cameroon, univ-ndere.cm

**Keywords:** chemical composition of yoghurts, native and modified starches, physicochemical properties of yoghurt, rheological properties of yoghurts, sensory properties of yoghurts, yoghurt with added starch

## Abstract

Reduced fat yoghurt is the most consumed type of yoghurt in the world, especially in developed countries. The shared opinion according to which it is better for health than plain yoghurt does not have a scientific basis. Many cohort studies instead suggest that full fat dairy products intakes have an inverse association with obesity and overweight. Most studies (more than 90%) have studied the effects of the addition of starch to milk on the characteristics of yoghurts instead of the effect of the replacement of milk fat by starch on these characteristics. When compared with control yoghurts made without adding starch, the addition of starch increases the carbohydrate content of yoghurts while decreasing the moisture content. There is not a specific trend with respect to mineral content, fat content, and protein content of yoghurt since an increase, a decrease or no effect are observed. The addition of starch increases the pH and apparent viscosity of yoghurts and decreases yoghurt syneresis, the effect being more important with increasing concentration of starch. The effects of the addition of starch on textural and sensory characteristics of yoghurts depend on the range of concentrations of starch used. Optimum sensory characteristics of yoghurts with added starch seem to be reached at a concentration of 2%, regardless of the source and eventual modification of starch. During the storage of yoghurts, there is a continuous decrease of pH and increase of lactic acid due to the increase of the population of lactic bacteria. The variation of apparent viscosity of yoghurts, their susceptibility to syneresis and their textural characteristics are dependent on the range of concentrations of starch used. No significant variation of sensory characteristics of yoghurts with added starch is observed over time. The addition of starch to yoghurts does not change their fundamental rheological characteristics.

## 1. Introduction

Yoghurt (which is also written yogurt) can be defined as a coagulated milk product, containing at least 10^7^ CFU/mL of lactic acid bacteria, which has a smooth texture and mildly sour taste and flavor, and which is obtained by fermentation of milk using lactic acid bacteria ([1–7]). It is the oldest safe and most popular fermented milk product in the world [8]. The world market of yoghurt is expected to grow annually at the rate of 5.99% and was estimated to be $144.29 billion in 2025 [9]. Although the exact origin of fermented milk products is difficult to ascertain, they likely date back to more than 10,000 years old [10]. Many countries in the world claim yoghurt as their own invention, though there is no clear evidence as to where it was first discovered [11]. Nomadic people used to preserve milk in containers made from stomachs of animals and it resulted in a dense and acidic food [12]. What is sure is that the first written mention of yoghurt was done in a Turkish book in 1070 BCE [11]. Although the consumption of dairy products remained the same from the end of 20th century until now, the overall capita consumption of yoghurt has doubled in the same period (at least in the Unites States) [13]. This increasing consumption of yoghurt can be explained by its nutritious and numerous health benefits. These health benefits include improving heart health (due to reduced LDL and VLDL cholesterol); weight loss; reduced blood pressure of hypertensive patients; improved insulin sensitivity and glucose tolerance; decreased risk of osteoporosis; improved digestion of lactose by lactose‐intolerant individuals (due to probiotics); boosting of the immune system; prevention of constipation; treatment of diarrhea, inflammatory bowel disease, and irritable bowel disease (due to probiotics); and treatment of allergy [14–16]. Yoghurt, in addition to the nutritious properties of milk, contains a relatively important amount of potassium, free peptides, and free amino acids (especially histidine, valine, and serine) [10, 11]. Since the works of Elie Metchnikoff in the 1900s, yoghurt is defined as being made from *Lactobacillus delbrueckii* subsp. *bulgaricus* and *Streptococcus salivarius* subsp. *thermophilus* [10, 12]. Yoghurt can be made using fresh milk or reconstituted powdered milk [17]. Fresh milk is mostly used at large scale in developed countries since the production of milk is very important there, whereas in developing countries, although yoghurt is sometimes produced from fresh milk (to those who have access to it), it is mostly made at artisanal and industrial scale from reconstituted powdered milk.

Several criteria are used to classify the different types of yoghurt that exist on market shelves. In this respect, yoghurts can be classified according to their manufacturing methods (set yoghurt, stirred yoghurt, and drinking yoghurt), their style (white yoghurt, dessert‐type yoghurt, and enriched yoghurt), and their chemical composition/fat content (full‐fat/plain, reduced fat, and low‐fat) [14, 15, 17–19]. Beyond these classifications, in some countries like Turkish, commercial yoghurts are enriched with plant protein [20] or with plant extracts, which have antioxidant properties [21, 22]. Set yoghurt, stirred yoghurt, and drinking yoghurt have a jelly‐like, semisolid, and fluid‐like texture, respectively [12]. Set yoghurt is incubated and cooled in the container to form a continuous and undisturbed gel structure, whereas stirred yoghurt is made by incubating the yoghurt in tanks, followed by stirring, cooling and packaging [12]. Drinking yoghurt is a stirred yoghurt that has a total solids content not exceeding 11% and which has undergone homogenization to further reduce the viscosity [11]. Based on their style, white yoghurts are made from milk with possible addition of milk cream; dessert‐type yoghurts are made with added foodstuffs (fruit pieces, cereals, cocoa, malt, chocolate, royal jellies, honey, and coffee); and enriched yoghurts are made with nutrients, compounds, or microorganisms with identified health properties (vitamins, oligosaccharides, fibers, probiotics, or other functional ingredients) [12]. Beyond these types of yoghurts, other regionalized or localized types exist like Greek‐yoghurt, Balkan‐style yoghurt, and French‐style yoghurts for instance [12]. Full‐fat yoghurt is made from full‐fat milk, whereas reduced fat and low‐fat/nonfat yoghurts are made from reduced fat and low‐fat milk, respectively [12]. Full‐fat/plain yoghurt is made from milk with at least 3.25% of milk fat [23, 24]. Low/reduced fat yoghurts are made from milk with 0.5%–2% [23] or from milk that has a fat content lower or equal to 1.5% [24]. Nonfat yoghurt is made from milk, which has a fat content of less than 0.5% [23]. Consumption of full‐fat dairy products is by far less appreciated by consumers, especially in developed countries, due to the shared opinion according to which milk fat, which is mostly made from saturated fatty acids, is associated to negative health effects of these fatty acids [25–27]. In fact, milk of animals (ovine, bovine, and caprine) contains 55%–75% of saturated fatty acids, 22%–39% of monounsaturated fatty acids, 2.4%–7.3% of polyunsaturated fatty acids, and 0.2%–2.4% of conjugated linoleic acid [28]. Food safety authorities of many countries and regional organizations (like the European Union) and the World Health Organization recommend to reduce intake of saturated fat as low as possible and/or to maintain their contribution to less than 10% of total energy intake [29]. These recommendations are based on observational nutrition studies done during the 1960s–1970s that showed a direct association between saturated fat consumption, raised blood cholesterol, and increased risks of cardiovascular diseases [29]. Many recent reviews of cohort studies done in many countries throughout the world [29–31] clearly show that there is a neutral or inverse relationship between the intake of dairy products, cardiovascular diseases, and cardiovascular heart diseases risks, regardless of the fat content of the dairy product. In the case of fermented milk products like yoghurt or cheese, many cohort studies have shown that full‐fat and low‐fat products produce similar positive effects on body composition, blood lipid profile, and cardiometabolic disease risk [29]. In some cases, it was observed that fermented milk products reduce mortality and risks of hip fracture, whereas the reverse is observed when milk is consumed [29]. These positive effects have partly been attributed to the increase of microbiota‐related metabolites (especially short‐chain fatty acids) such as butyrate, hippurate, and malonate, which are not found or produced at very limited extent when milk is consumed [29]. Contrary to what is commonly considered by yoghurt consumers, full‐fat yoghurt might have healthier effects than reduced fat and low‐fat yoghurt. It has been shown in a cohort study involving 114,682 adults that although yoghurt and full‐fat dairy intakes have an inverse association with overweight and obesity, skimmed, semiskimmed, and nonfermented dairy products had positive association with overweight and obesity [32].

It is this preference for reduced fat products that has motivated the development and large selling of reduced fat and low‐fat yoghurt, especially in developed countries. From a technological point of view, fat reduction in yoghurt is associated with many perceptible defaults of yoghurt that are poor texture, weak body, high syneresis/wheying off, relatively low firmness, as well as the reduction of many other sensory and organoleptic characteristics of yoghurt [7, 26, 33–37]. In order to correct these defaults, skimmed milk powder, casein‐based powders (like sodium caseinate), whey protein–based powders (like whey protein concentrates or isolates), and hydrocolloids are used with the major objective to improve the appearance and viscosity of yoghurts [34, 38]. Hydrocolloids used in yoghurt manufacture are carboxymethylcellulose, guar gum, xanthan gum, carrageenan, alginates, pectins, mucilages, glucomannan, gelatin, and starch (native and modified) [16, 16, 39–43]. Although these hydrocolloids are authorized and used in the manufacture of commercial yoghurts, those which are most commonly used, probably because of their relative abundance when compared with other stabilizers, are gelatin and starch [44, 45]. Plant‐based thickeners like starch are thought to be less expensive than milk based additives [16, 16, 44]. A number of literature reviews have been done on the use of hydrocolloids in yoghurt [14, 39, 41, 46]. Because of the number of hydrocolloids used in yoghurt manufacture and which deserve attention, these reviews considered starches used in yoghurt as a homogeneous group and no comparison was generally done between the types of starch used (which differ in terms of their source, the eventual modification done) nor their specific effects nor concentrations on the chemical composition, rheological, and sensory properties of yoghurt. The present review is aimed at covering these limits by specifically focusing on starch.

## 2. Methodology Used for the Review

This review is based on data available in published literature. Several databases were used, including Scopus, ScienceDirect, Google Scholar, ResearchGate, and Wiley Online Library. The keywords used for the search included “Yoghurt,” “Starch,” “Fat substitution of yoghurt,” “Fat replacement in yoghurt,” “Effect of starch on yoghurt,” “Starch addition in yoghurt,” “Rheological properties of yoghurt with starch,” and “physico‐chemical properties of yoghurt with starch.” Once a paper was obtained, the reference list of each paper was also consulted in order to download relevant papers for the topic of interest. The different information found in each paper was gathered, and once all the information from all the documents consulted was obtained, it was organized in the different sections for which information was available.

**Figure 1:**
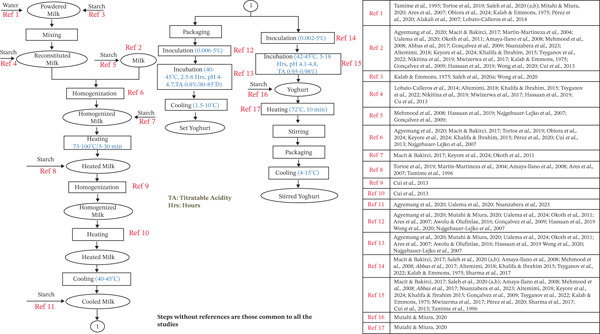
Processes used for yoghurt production when using starch.

In most studies done on yoghurt to which starch was added or used to substitute fat, the one made without starch or with lesser amount of fat is always used as reference for comparison purposes. Since yoghurt from different studies is made from milk with different fat and protein content, using different fermentation conditions, made with different concentrations and origin of starter cultures, and using starch obtained sometimes from different plants and different processing conditions (Tables 1, 2, and Figure 1), it is not possible to consider the yoghurt of a given study as the reference for all the studies. However, in each study, generally, only the concentration of starch changes between the different samples. This offers the possibility (for studies where values are provided) to calculate the variation (in percentage) from reference yoghurt for each characteristic (chemical composition, physicochemical properties, rheological, and sensory characteristics). By proceeding like that for all the studies, the same scale (variation in percentage) can be used across studies. In the case of a reduction of the value of a given characteristic with addition of starch/substitution of fat when compared with reference yoghurt, a negative variation is obtained, and a positive variation in the case that there is an increase of the value of a given characteristic. A null variation shows that there is no effect of the factor under consideration. It is through the use of this methodology that variation percentages of the effect of the concentration, source, and modification of starch on the chemical composition, physicochemical and rheological properties of yoghurt and on microorganism growth (Effect of concentration, Effect Conc. and source, and Effect Conc. and modification spreadsheets of the Supporting Information S1), The effects of duration time on the chemical composition, physicochemical, and rheological properties of yoghurt, and on microorganism growth (Effect Conc. and day, Effects of time, Effect time and modification, and Effect time and concentration spreadsheets of the Supporting Information S1); and the effect of the concentration and modification of starch on sensory characteristics (Sensory evaluation spreadsheet of the Supporting Information S1), rheological model (Rheological model spreadsheet of the Supporting Information S1), and parameters (Rheological parameters spreadsheet of the Supporting Information S1) of yoghurt were obtained by gathering data collected from the different documents used for the review. All the graphs of this review have been done using the software SigmaPlot 12.0.

**Table 1:** Sources, concentrations, and modifications of starches used in yoghurt production.

Source of starch	Modification	Production and modification of starch	Concentration	Form of addition	Authors
Cassava	Native	Produced by authors.	2.5%, 5%, 7.5% (*W*/*V*)	Solid form.	[33]
Cassava	Native, acetylation, cross‐linking	Bought.	No precision	Addition of 1% starch solution.	[26]
Cassava and taro	Gelatinization	Produced by authors.	0.5%, 1% (*W*/*V*)	A pregel was added.	[43]
Cassava	Native, enzymatically modified	Enzymatic modifications done by authors.	1% (*W*/*V*)	Solid form.	[47]
Cassava and maize	Pregelatinization of cassava starch	Maize starch was bought and cassava starch produced by authors.	0.1%, 0.5%, 0.6%, 1% (no precision)	Solid form.	[48]
Cassava, maize and waxy maize	Native, cross‐linking (cassava and waxy maize)	Bought.	1% (*W*/*V*)	Solid form.	[13]
Cassava and maize	Native	Produced by authors.	No precision	No precision.	[49]
Yam (*Discorea alata*)	Native	Produced by authors.	0.5%, 1%, 1.5%, 2%, 3% (*W*/*V*)	Solid form.	[8]
Yam (*D. alata*) and cassava	Native, acetylation, oxidation, hydrolysis (acid thinning)	Modifications done by authors for yam and bought for cassava (native).	2%	Solid form.	[50]
Yam (*D*. *alata* and *Discorea rotundata*)	Native	Produced by authors.	No precision	5% gel was added.	[51]
Potato, sweet potato, chickpea beans, Turkish bean, maize	Native	Production done by authors, except for corn starch, which was bought.	0.1% (*W*/*V*)	Solid form.	[35]
Potato, sweet potato, chickpea beans, Turkish bean, maize	Native	Production done by authors, except for corn starch, which was bought.	1% (*W*/*V*)	Solid form.	[36]
Potato, maize, waxy maize, cassava	Native	Bought.	1.5% (no precision)	No precision.	[52]
Sweet potato	Native	Production done by authors.	0.25%, 0.5%, 0.75%, 1% (no precision)	Solid form.	[18]
Potato	Native, enzymatically modified	Enzymatic modifications done by authors.	2% (*W*/*V*)	Solid form.	[34]
Taro	Native	Production done by authors.	0.5%, 1%, 1.5%, 2%, 2.5%, 3% (*W*/*V*)	Solid form.	[16, 16]
Cocoyam	Native	Production done by authors.	10% (*W*/*V*)	Solid form.	[17]
Ensete and maize	Native, alcoholic alkaline	Production made by authors for ensete; maize starch was bought.	1%, 2%, 3% (*W*/*V*)	Solid form.	[25]
Wheat	Esterification	Modification done by authors.	No precision	No precision.	[53]
Pearl millet	Esterification	Production of starch and modification done by authors.	0.5%, 1%, 1.5%, 2% (*W*/*V*)	Solid form.	[54]
Maize	Modified (no precision)	Bought.	0.3%, 0.5% (*W*/*V*)	Solid form.	[42]
Maize	Native	Bought.	0.1%, 0.2%, 0.3%, 0.4%, 0.5% (no precision)	No precision.	[7]
Maize	Native	Bought.	1.25% (*W*/*V*)	Solid form.	[55]
Maize	No precision	Bought.	0.5%, 0.75%, 1% (no precision)	No precision.	[3]
Waxy maize	Cross‐linking	Bought.	0.1%, 0.15%, 0.2% (*W*/*W*)	Solid form.	[56]
Waxy maize	Pregelatinization	Bought.	2% (*W*/*V*)	Solid form.	[57]
No precision	Oxidation and hydroxypropylation	Bought.	0.1%, 0.5%, 1% (*W*/*W*)	Solid form.	[58]
No precision	Oxidation and hydroxypropylation	Bought.	0.1%, 0.5%, 1%	No precision.	[59]
No precision	Cross‐linking	Bought.	1%, 2%, 3%, 4%, 5% (*W*/*W*)	Solid form.	[60]
No precision	No precision	Bought.	0.5% (no precision)	No precision.	[61]
No precision	No precision	Bought.	2.79%, 3.72%, 4.65% (*W*/*V*)	Solid form.	[27]
No precision	Cross‐linking	Bought.	0.5%, 1%, 1.5% (*W*/*W*)	Solid form.	[62]
No precision	No precision	Bought.	1.5% (*W*/*W*)	Solid form.	[24]

*Note:* Cassava (*Manihot esculenta* Crantz), taro (*Colocasia* spp.), cocoyam (*Colocasia esculenta*), maize (*Zea mays*), yam (*Discorea alata* and *Discorea rotundata*), potato (*Solanum tuberosum*), sweet potato (*Ipomea batatas*), chickpea beans (*Cicer arietinum*), Turkish bean (*Phaseolus vulgaris* L.), wheat (*Triticum* spp.), ensete (*Ensete ventricosum*), and pearl millet (*Pennisetum typhoides*).

**Table 2:** Types of yoghurts, characteristics of milk, and yoghurt production parameters used for yoghurt production.

Type of yoghurt	Milk used	Total solids of milk (g/100 mL)	Heat treatment parameters	Inoculation characteristics	Incubation parameters	Cooling	References
Stirred yoghurt	Whole, partially, and fully skimmed milk	No precision	85°C–92°C, 10 min	2.5% (*W*/*V*)	42°C–45°C, 5–6 h	10°C	[33]
Set yoghurt	No precision	No precision	80°C, 20 min	0.02% (*W*/*V*)	44°C, pH = 4.7		[55]
Set yoghurt	Powdered cow milk	No precision	85°C–90°C, 5 min	2.5% (*W*/*V*)	42°C–45°C, 4–5 h	4°C	[8]
Set yoghurt	Cow′s milk (2.5% fat)	No precision	85°C, 20 min	0.006% (*W*/*V*)	45°C, 2.5 h	5°C–6°C	[53]
Set yoghurt	Skimmed cow′s milk	No precision	No precision	2% (no precision)	44°C, pH = 4.7	4°C	[52]
Set yoghurt	Powdered skimmed milk	14 (including starch)	60°C, 30 min	0.042% (*W*/*V*)	42°C, pH = 4.6		[35]
Stirred yoghurt	Powdered skimmed milk	12 (not including starch and including sugar added at 6% *W*/*V*)	90°C, 5 min	0.002% (*W*/*V*)	43°C, 5 h, pH = 4.6 until 0.95%–0.98% TA	5°C	[26]
Stirred yoghurt	Cow′s milk	No precision	90°C, 20 min	5% (no precision)	42°C, 18 h	4°C	[43]
Stirred yoghurt	Cow′s milk	(Sugar was added at 6.5% *W*/*V*.)	90°C, 30 min	0.02% (no precision)	45°C, 5 h, pH = 4.1–4.4	5°C–10°C	[42]
Set yoghurt	Milk (2.5%)	13 (including starch)	85°C, 20 min	1.5*%*LB + 0.2*%*ST (no precision)	45°C, 3 h or pH = 4.5	4°C	[40]
Stirred yoghurt	Powdered skimmed milk	(Sugar was added at 8% *W*/*V*.)+	90°C–95°C, 15 min	0.002% of a 0.264% prepared starter culture	42°C, pH = 4.5	5°C	[58]
Stirred yoghurt	Skimmed milk	11.1 (not including starch and sugar added at 8%)	90°C–95°C, 15 min	No precision	42°C, pH = 4.5	5°C	[59]
Stirred yoghurt	Powdered milk	(Sugar was added at 10% *W*/*V*.)	90°C, 15–30 min	0.05% (*W*/*V*)	44°C, 7 h, pH = 4.2	1.5°C	[50]
Set yoghurt	No precision	No precision	73°C, 15 min	2% (no precision)	42°C, 0.8% TA, pH = 4.25	6°C	[7]
Set yoghurt	Buffalo′s milk (1.5% fat)	No precision	90°C, 5 min	2% (*W*/*V*)	45°C, 3 h	4°C	[56]
No precision	Powdered milk	No precision	95°C, 15 min	2% (no precision)	45°C, 8 h	5°C	[17]
Set yoghurt	Cow milk	No precision	90°C	No precision	40°C, 12 h	15°C	[49]
Set yoghurt		No precision	90°C, 30 min	3% (*W*/*V*)	42°C, 4 h	5°C	[18]
Set yoghurt	Defatted cow milk (1.5%)	13.4 (not including starch and including sugar added at 2% *W*/*V*)	85°C, 30 min	No precision	42°C	5°C	[25]
Set yoghurt	Whole camel milk	No precision	90°C, 10 min	2% (no precision)	42°C, pH = 4.5–4.6	No precision	[60]
Set yoghurt	Skimmed milk	No precision	100°C, 30 min	5% (*V*/*V*) using 0.1% starter culture (yoghurt) fermented at 37°C, 16 h	40°C, 8 h	4°C–5°C	[47]
Set yoghurt	Powdered skimmed milk	14 (including starch)	60°C, 30 min	3% (*W*/*V*)	42°C, pH = 4.6	5°C	[36]
Set yoghurt	Powdered skimmed milk	(Sugar was added at 3.4% *W*/*V*.)	88°C, 15 min	2% using yoghurt culture	44°C, pH = 4.6	4°C–5°C	[57]
Set yoghurt	Low‐fat milk (1.5%)	11 (not including starch)	90°C, 15 min	No precision	42°C, 4 h	4°C	[48]
Set yoghurt	Powdered skimmed milk	12 (not including starch)	No precision	No precision	42°C, pH = 4.5	4°C	[51]
Set yoghurt	Low‐fat milk (1.5%)	No precision	No precision	2% (*W*/*V*) using a starter culture (yoghurt) incubated at 37°C, 12–14 h	40°C, pH = 4.5	4°C	[54]
No precision	No precision	No precision	No precision	5% (*V*/*V*) using a starter culture (yoghurt) incubated at 37°C, 16 h	37°C, pH = 4.5–4	No precision	[34]
No precision	No precision	No precision	80°C, 20 min	No precision	45°C, 16 h	No precision	[3]
Stirred yoghurt	Buffalo′s milk	17.1 (not including starch, sugar was added at 8% *W*/*V*.)	90°C, 10 min	2% prepared starter culture	42°C until coagulation		[61]
Stirred yoghurt	Powdered milk	No precision	91°C, 30 s	0.015% (*W*/*W*)	43°C, pH = 4.6	Less than 10°C	[27]
Set yoghurt	Powdered skimmed milk	25 (not including starch)	95°C, 30 min	No precision	40°C, 6 h	4°C	[62]
Set yoghurt	Skimmed milk and full‐fat milk	(Sugar was added at 6% *W*/*V*.)	85°C, 15 min	0.3% (*W*/*V*)	45°C, up to 80–85°D	4°C	[13]
Set yoghurt	Powdered skimmed milk	14 (not including starch)	90°C, 5 min	No precision	42°C, 6.5 h	No precision	[24]
Set yoghurt	No precision	No precision	No precision	2%	44°C, pH = 4.7	4°C	[52]

Abbreviations: LB, *Lactobacillus delbueckii* subsp. *bulgaricus*; ST, *Streptococcus thermophiles;* TA, titratable acidity.

## 3. Diversity and Uses of Starches in Yoghurt

**Figure 2:**
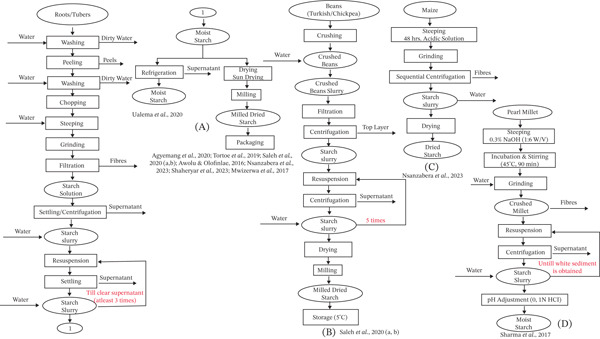
Processes used for the extraction of starches used in yoghurt production. (A) Roots and tubers, (B) legumes, (C) maize, and (D) pearl millet.

Starches used in yoghurt production in literature are from roots and tubers (cassava, yam, potato, sweet potato, taro, ensete, and yam), legumes (Turkish beans and chickpea beans), cereals (wheat, maize [nongenetically modified and waxy]), and pseudocereal (millet) (Table 1), although many authors did not mention the plants from which starches were obtained [24, 27, 58, 60–62]. Considered individually, the most important plant parts from which starches were extracted are maize/waxy maize seeds (≈34% of plants used) and cassava roots (≈20% of plants used) (Table 1). These starches were mostly bought (≈64% of studies), but also produced by authors (≈36% of studies) (Table 1 and Figure 2). The importance of maize starch can be explained by the fact that it represents 80% of the world market production of starch [63]. In sub‐Saharan African countries, it is the most available starch found on market shelves, although it is generally imported.

Roots, tubers, and legumes starches used in yoghurt production were extracted in aqueous solutions through many steps including the grinding and sieving of these plant parts (Figure 2). Maize starch was extracted after steeping in an acidic aqueous solution [49], whereas pearl millet starch was extracted after steeping in alkaline aqueous solution and was then neutralized using an acidic solution [54]. Starches used were either native or chemically modified (esterification, acetylation, cross‐linking, oxidation, hydrolysis, hydroxypropylation, and alcoholic alkalinization), physically modified (gelatinization), or enzymatically modified (partial hydrolysis) (Table 1). These modifications were rarely done by authors (Table 1). Native and chemically modified starches were mostly used in studies (≈46% and ≈38%, respectively) (Table 1). In fact, native starch, which is rich in Type 2 resistant starch, is insoluble in water at room temperature and resistant to hydrolysis by amylase [63, 64]. The modifications of starches are intended to improve some or many of their functionalities like their high viscosity at low temperature (temperature used to preserve yoghurt, for instance) or their ability to withstand low pH conditions (found in yoghurt) [63, 65]. Modifications of starches lead to reduced quantities needed to obtain effects, which are similar to native starch.

Starch can be modified chemically, physically, genetically, and enzymatically [63]. The different chemical modifications of starch are hydrolysis (acidic and alkaline), esterification (acetylation, succinylation, and phosphorylation), etherification (hydroxypropylation, hydroxyethylation, and carboxymethylation), oxidation, and cross‐linking [63, 65, 66]. Cross‐linking may employ hydrolysis, oxidation, esterification, etherification, phosphorylation, a combination of these methods in a sequential, or one‐mix procedure [63]. Chemically modified starches can also be classified as converted starches (which include oxidized starch, acid, and alkaline‐treated starches), stabilized/substituted starches (which are obtained through esterification or etherification), and dextrinized starches [67]. Physical modifications of starch involve pregelatinization, ball milling (stirring and vacuum), annealing, osmotic pressure treatment, heat‐moisture treatment, dry heating, superheating, production of granular cold water soluble starch, multiple deep freezing and thawing, instantaneous controlled pressure‐drop process, pulse electric field treatment, and corona electric discharge [63, 67]. Due to the fact that chemicals are not used during physical modifications of starches, they represent the cheapest, simplest, and cleanest modification methods [63]. In the literature, amongst the physical modifications of starches, only gelatinization has sometimes been used [43, 48], especially in Africa. Africa is diverse in terms of starchy foods from different plants that are processed and consumed from childhood to adult life [68, 69]. Because of potential health concerns associated with genetically and chemically modified starches as well as the cost associated with enzymatically modified starches, consumers (mostly from developed countries) are more oriented to native and physically modified starches. Taking into account the important diversity of starchy plants found in Africa, the advantages associated with physically modified starches, and the fact that sub‐Saharan African countries are the poorest countries of the world [70], studies of the effects of physical modifications of starches from these plants offer an important venue for future research.

The addition of starch was always done once and at one specific step, either directly to the raw material used (powder milk or liquid milk) or after homogenization or after pasteurization or to cooled milk before inoculation and incubation or after incubation/fermentation of milk to produce yoghurt (Figure 1). Starch added during yoghurt production was mostly in solid form (≈69% of studies) but also in the form of gel [43, 51] or starch solution [26] (Figure 1 and Table 1). Concentrations used in literature were varying between 0.1% and 7.5% (2.29% on average) (*W*/*V*) or between 0.1% and 5% (1.44% on average) (*W*/*W*) (Figure 1). From average concentrations, it is clear that concentrations used rarely exceed 2% (*W*/*W* or *W*/*V*) (Table 1). Since yoghurt is made from liquid milk and that starch is mostly added in a solid form (Figure 1 and Table 1), concentrations of starch added to milk are mostly expressed in *W*/*V* (≈66% of studies) (Table 1). It is worth noting that some studies neither mention the form (solid or gel) in which starch was added to yoghurt nor the concentrations of starches that were used in their studies (Table 1). Regardless of the step where starch was added, it is always followed by heating (mostly pasteurization) at 72°C–100°C for 5–30 min (Figure 1 and Table 2). These conditions are known to lead to different degrees of gelatinization of starches that are added to yoghurt, probably leading to different effects on rheological and sensory properties of yoghurt. Cooled gelatinized starch, as it probably occurs in chilled yoghurt, is known to be rich in Type 3 resistant starch [64] and therefore, the consumption of yoghurt enriched with starch might produce the nutritional advantages associated with the consumption of insoluble fibers like their antihyperlipidemic, antidiabetic, and anticancerous effects [71].

## 4. Characteristics of Yoghurts Produced Using Starches and Approaches to Optimize the Quality of Yoghurt

Yoghurt produced in studies involving starches are mostly set yoghurts (≈71% of studies) and few interest was given to stirred yoghurt (≈29% of studies) (Table 2). This seems obvious since syneresis, which can be defined as the shrinkage of gel, and which leads to whey separation or the expulsion of interlocked whey (serum) from the continuous yoghurt network [35, 36, 39, 72, 73], is a very important defect, which is easily and mostly observed in set yoghurts made from low‐fat milk. These studies have mostly involved skimmed milk (≈68% of mentioned type of milk used), but also full‐fat milk (≈32%) (Table 2). Although cow′s milk is the most used, buffalo and camel milks were also used (Table 2). Yoghurts produced using starches were mostly unsweetened, and when sugar was added, concentrations used were varying between 2% and 10% (*W*/*V*) (Table 2). Parameters used for pasteurization were 73°C–100°C/5–30 min (average of 87°C/17 min) (Table 2). Heating of milk is known to induce whey protein denaturation (*β*‐lactoglobulin mostly), which can thus associate to casein micelles [46]. Heating also reduces the gelation duration (through an improvement of the viscosity), syneresis, and increases the pH at gelation [46, 74, 75]. The best conditions are 85°C/30 min or 90°C/5 min [74, 76]. From what is observed, most authors have used these optimum conditions (Table 2).

Milk is mostly inoculated with microorganisms in their solid form (Table 2), but sometimes with a previously prepared starter culture (another yoghurt) [47, 54, 57, 58, 61]. This approach has the potential to reduce the fermentation duration since microorganisms are already in their living form while in their solid form, microorganisms will spend some time in the milk in order to be revivified before effectively carry out the fermentation process. In sub‐Saharan African countries, since lyophilized microorganisms are not easily found in markets, yoghurt is generally made using previously produced batch. There is a huge variation (up to 200 times) of concentrations of microorganisms used for fermentation. Concentrations used are 0.006%–5% (1.56% in average) for set yoghurt and 0.002%–5% (0.85% in average) for stirred yoghurt (Table 2 and Figure 1). When using prepared starter cultures (yoghurts), concentrations low as 0.002% of a prepared yoghurt incubated using microorganism concentration of 0.264% can be effective for fermentation. Although lyophilized lactic bacteria sold have different concentrations of these bacteria (even from the same supplier), neither these concentrations were supplied nor the effective concentration of lactic bacteria when previously prepared starter cultures were used. Future studies should therefore provide the concentration of starter culture used or of lyophilized microorganisms used. According to the Codex Alimentarius, the minimum quantity of microorganisms of the starter culture is 10^7^ CFU/g ([4]). The addition of sugar during yoghurt production can delay microorganism growth and acid production (probably because of the osmotic effect of sugar) as well as impact the flavor of yoghurt. It has been shown that yoghurts with sugar content of 4% have reduced growth of microorganisms and thus a reduced acid production, and beyond 8% the production of acetaldehyde is highly impacted [77].

Fermentation conditions are 40°C–45°C (average of 42.6°C)/3–8 h (average of 5.35 h) for set yoghurt and 42°C–45°C (average of 42.9°C)/5–18 h (average of 8.1 h) for stirred yoghurt (Table 2 and Figure 1). Considering both types of yoghurt, the average fermentation temperature when using starch is 42°C, and the average duration is 5 h and 30 min (by neglecting atypical long fermentation durations like 18 h). It is worth noting that the reduction of fermentation temperature is associated to an increase of fermentation duration (Table 2). Although fermentation duration is used to stop fermentation, the pH of yoghurt or its titratable acidity are most used parameters. In this respect, fermentation ends when the pH is between 4–4.8 and/or the titratable acidity between 0.8%–0.98%/80–85^0^D (Table 2 and Figure 1). The final concentration of lactic acid in yoghurt is known to vary between 0.7%–1.2% [11]. Lactic acid bacteria used are mostly *Streptococcus thermophilus* and *L*. *delbrueckii* subsp. *bulgaricus*. Although the first mostly contribute to the production of compounds which contribute to yoghurt aroma (formate, acetaldehyde, acetoin, acetone, and diacetyl), the second mostly contribute to the production of lactic acid, which will allow precipitating caseins [78, 79]. However, some authors affirm that individually, each microorganism contributes to the production of flavor compounds and acid at different extent and when mutualized, the production of these compounds is more important [11, 12]. In the literature, 42°C is the optimal temperature to use for yoghurt fermentation [78, 79]. The reduction of fermentation temperature to 38°C–40°C, although it is associated to longer fermentation duration, is known to improve gel firmness and viscosity, and to reduce whey separation [72, 75, 80]. Based on the work done by some authors [81], it seems that fermentation at different temperatures during the same batch can allow achieving better textural properties. In Africa, because of the absence of fermenters to maintain constant temperature and/or sometimes the scarcity of light in many localities where yoghurt is commonly produced, fermentation is carried out in containers (flasks and pots) that are covered with blanket or any other material, which can decrease the quick drop of temperature. These fermentation conditions lead to a progressive decrease of temperature, producing a temperature gradient that has the potential to optimize the textural properties of yoghurts. These traditional approaches of fermentation deserve to be studied in order to better understand their impact on yoghurt quality.

## 5. Effects of the Addition of Starch on Characteristics of Yoghurts

The effects of the addition of starch have been evaluated on the chemical composition, physicochemical properties (pH and titratable acidity), apparent viscosity and syneresis, rheological properties, and sensory properties of yoghurts (Supporting Information).

### 5.1. Effect of the Addition of Starch on the Chemical Composition of Yoghurt

The effect of the addition of starch has been assessed on protein content, fat content, carbohydrate content, ash content, and moisture content of yoghurts (Supporting Information, Effect of concentration).

When compared with reference yoghurt, the addition of starch either decreases (3.5%–20%) [3, 16, 16, 61] or increases (1.2%–15.3%) [47, 55] the ash content of yoghurts (when compared with yoghurts prepared without using starch). When considering yoghurts prepared using different concentrations of starch [3, 16, 16], there is not a specific trend, which might be an indication of the fact that starch might not be uniformly distributed in yoghurt or that water is differently trapped in yoghurts at different concentrations of starch.

When compared with reference yoghurts, the addition of starch either reduces (0.6%–18%) [3, 13, 16, 16, 55, 57, 61] or increases (1.6%–13%) [16, 16, 34, 47] or has no effect [34, 49] on the protein content of yoghurts. When maize starch is used, increasing the concentration of starch decreases the protein content of yoghurts (when compared with the control) [3]. It is worth noting that when taro starch is used, there is not a specific trend when the concentration of starch is increased [16, 16].

The addition of starch either reduces (0.36%–50%) [13, 34, 55, 61], increases (0.4%–15%) [3, 16, 16], or has no effect on the fat content of yoghurts [3, 61]. The highest reduction (50%) [13] can be explained by the fact that the control used in that specific study was a full fat yoghurt, whereas the yoghurts to which starches were added were low‐fat yoghurts.

The addition of starch increases the carbohydrate content (8.5%–43.9%) [34, 47, 61] and total solids of yoghurts [34], the increase being more important with increasing concentration of starch.

The addition of starch generally reduces (0.1%–9.6%) the moisture content of yoghurts [13, 16, 16, 61]. Increasing the concentration of starch decreases the moisture content of yoghurt, apparently up to 2% [16, 16].

Starch is a carbohydrate and therefore, its addition during yoghurt production is associated with an increase of carbohydrate content as observed, concomitantly with an apparent decrease of other components, which is really due to an increase of the total dry matter. Because of its composition, the addition of starch is supposed to increase the carbohydrate content and to reduce fat content, protein content, lipid content, ash content (which indirectly determine minerals), and moisture content as observed in many studies.

### 5.2. Effect of the Addition of Starch on pH and Titratable Acidity

**Figure 3:**
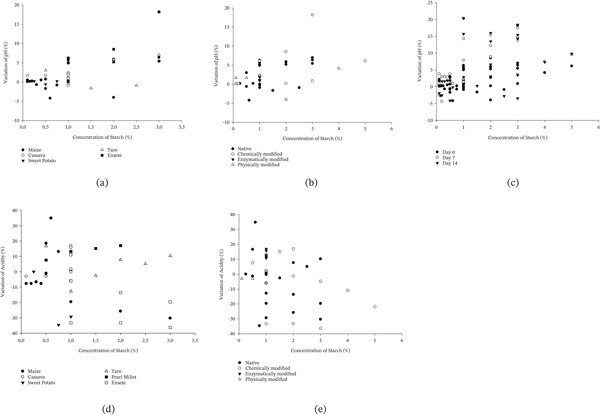
Effects of the addition of starch on the variation of acidity and variation of pH of yoghurts. (a) Effect of the origin of starch on the variation of pH of yoghurt, (b) effect of the modification done on starch on the variation of pH of yoghurt, (c) effect of day of analysis on the variation of pH of yoghurt, (d) effect of the origin of starch on the variation of acidity of yoghurt, and (e) effect of the modification done on starch on the variation of acidity of yoghurt [3, 7, 13, 16, 16, 18, 25, 47, 48, 54, 56, 57, 60, 61].

Regardless of the factor considered (source of starch, modification done, and day of analysis) (Figure 3), when compared with reference yoghurt, the addition of starch is generally associated with an increase of the pH of yoghurts (0.4%–27.2%) (Figure 3a–c; Supporting Information, Effect of concentration). When a decrease of pH is observed, it generally does not exceed 5% (Figure 3) and is independent of the source of starch (a decrease is observed for maize, waxy maize, taro, and cassava starches [3, 16, 16, 47, 48, 56]) and the eventual modification done on starch (a decrease is observed for native starches, cross‐linked starches, and enzymatically modified starches [16, 16, 47, 48, 56]). The increase of pHs (in comparison with reference yoghurt) is associated with a decrease of titratable acidity, the reverse being observed with a decrease of pHs (Figure 3d,e Supporting Information, Effect of concentration).

When considering the source of starch, the highest increase of pH is observed with ensete starch, whereas the highest decrease is observed with maize starch (Figure 3a). When considering the modification done on starch, native starch generally produces highest variation of pH and although an important variation is observed with chemically modified starches, it is in one study and is due to the fact that ensete starch is modified using alkaline (Figure 3b; Supporting Information, Effect of concentration). When considering the day when analyses of pH are done (Figure 3c), it is clear that highest variations (when compared with reference yoghurt) are observed when pHs are determined 7 and 14 days after production. This shows that there is pH variation (in comparison with reference yoghurt) with increasing storage duration and that this variation might be more important for samples with added starch.

Increasing the concentration of starch increases the variation of pH (in comparison with reference yoghurt) and this is independent of the source of starch and the modification done (Supporting Information, Effect of concentration) [16, 16, 18, 25, 48, 56, 60]. The production of lactic acid during yoghurt production is due to lactic bacteria, and higher pHs, which are associated with lower titratable acidity (Figure 3 and Supporting Information, Effect of concentration), might traduce the fact that increasing the concentration of starch decreases the activity of microorganisms (when compared with reference yoghurt). Instead, it is the contrary which seems to be observed, and increasing the concentration of starch increases the activity/quantity of lactic bacteria [25, 54]. This observation can only be explained by the fact that higher pHs (resulting from the addition of starch) favor the growth of microorganisms, which are thus less inhibited by the acidic medium, highest pHs being associated with more important growth of lactic bacteria. The source of starch as well as the modification done also have their effects. In this respect, it has been shown that modified ensete starch has the highest effect on lactic bacteria growth, then followed by native maize starch and finally native ensete starch [25]. The different variations of pH and titratable acidity between studies are probably due to the fact that different yoghurts were made using different initial loads of lactic bacteria.

### 5.3. Effect of the Addition of Starch on Apparent Viscosity and Syneresis of Yoghurts

Many authors have assessed the effect of the addition of starch on apparent viscosity [17, 33, 34, 43, 48, 50, 52, 54–56, 61] and syneresis of yoghurts ([13, 18, 26, 34, 48, 49, 56, 59, 61], [17, 35, 36, 43, 54]).

#### 5.3.1. Effect of the Addition of Starch on the Apparent Viscosity of Yoghurt

Apparent viscosity of yoghurts has been measured using different trademarks of viscometer, spindles with different sensitivities, at different speeds, and different temperatures. Temperatures used to measure the apparent viscosity of yoghurts are 25°C (≈40% of concerned studies) [35, 36, 56, 61], 20°C (≈10%) [34], and 4°C–8°C (≈50%) [16, 16, 25, 47, 48, 58]. These different conditions used make valid only intrastudies comparisons. Since yoghurts are stored at low temperatures like 4°C–8°C (Figure 2 and Table 2), it is advisable for future studies to measure yoghurt apparent viscosity at these temperatures in order to reflect the reality. Yoghurts with added starch have different apparent viscosities (Supporting Information, Effects of time). These differences probably result, beyond previously mentioned factors influencing the determination of apparent viscosity, from differences in total solids of milk used to manufacture these yoghurts (11%–25%) (Table 2).

Independently of the sources of starches and the eventual modification carried out on starches, the addition of starch generally increases the apparent viscosity of produced yoghurts (0.5%–179%) (when compared with reference yoghurts made without adding starch) [16, 16, 17, 25, 34, 43, 48, 50, 52, 61]. A decrease of viscosity was however reported by some authors, especially when using cross‐linked waxy maize starch (decrease by 5.6%–55.7% when compared with control yoghurt) [56], enzymatically modified potato starch (decrease by 3.6%–11% when compared with control yoghurt) [34], native taro starch (decrease by 0.3%–3.55% when compared with control yoghurt) [16, 16], and native maize starch (decrease by 8% when compared with reference yoghurt) [55]. For the study where higher decreases of the viscosity were observed [56], it can be explained by the fact that the control yoghurt is made from full fat milk (3% fat), whereas low‐fat milk (1.5%) was used for yoghurt samples with added starch. Neither the modification nor the source of starch are responsible for these decreases since the same modification and source of starch produce an increase of apparent viscosity when compared with reference yoghurts (Supporting Information, Effect of concentration). The decrease might traduce the fact that for yoghurt made with milk having the same total solids, the one with fat produces thicker yoghurts.

For studies in which different concentrations of starch were used [48, 54, 56], increasing the concentration of starch increases the apparent viscosity of yoghurts (when compared with control produced without starch). For the same source of starch, it seems to have a variety effect since starches produced from different varieties of yam have been shown to produce different apparent viscosity values for similar concentrations of starch [33]. When comparing native starches with modified starches, some authors [25] show that chemically modified starch allows obtaining yoghurt with higher apparent viscosity than native starch obtained from the same source (ensete). When comparing starches obtained from different sources, at similar concentrations, native corn starch produces more viscous yoghurts than native ensete starch [25]. When comparing modified starches from different sources (cross‐linked maize starch and cross‐linked cassava starch) at the same concentration, cross‐linked cassava starch allows obtaining yoghurts with lower viscosity than when using cross‐linked maize starch, although both yoghurts are more viscous than the one made using native maize starch [13]. These studies confirm that modified starches produce more viscous yoghurts than native starches. These studies seem to show that corn starches produce more viscous yoghurts than ensete and cassava starches.

In the study comparing cross‐linked cassava starch and cross‐linked maize starch, the modified starch from both sources was bought and their degree of cross‐linking not determined [13]. Such comparisons can only be reliable if starches from different sources are modified in such a way that they have the same degree of modification (which must be determined). Practically, it is difficult if not impossible because of the intrinsic differences, which exist between starches of different sources (polymerization degree of amylose and amylopectin, amylose/amylopectin ratio, and so on) and even between starches from the same source.

In order to determine the effect of starch on yoghurt viscosity, yoghurts are compared to a reference similarly prepared but without any added starch. Proceeding like that is known to increase the total solids content of yoghurt [34]. It has been clearly shown that for yoghurt made from milk only, increasing the total solids content allows increasing the apparent viscosity of yoghurt [72, 82]. Therefore, the increase of viscosity of yoghurt with increasing concentration of starch can be assimilated to an increase of the total solids content. In order to effectively understand the effect of addition of starch on apparent viscosity, the control yoghurt and samples with added starch must have the same total solids content. Although some authors have prepared their reference yoghurt in such a way that it was having the same total solids content as samples with added starch (by using powder milk, skimmed or not) [13, 27, 35, 36, 40, 50, 58], none of these authors compared the viscosity of the different samples (Complementary data, Effect of Concentration). Such approaches are welcome for future studies in order to really understand the effect of starch addition on yoghurt viscosity. It seems that optimum viscosity is obtained when milk with 14%–16% total solids content is used [82].

#### 5.3.2. Effect of the Addition of Starch on Yoghurt Syneresis

Syneresis, which is also known as wheying off, is serum expulsion from gel [35, 36, 39]. It is mainly found in set yoghurt although stirred yoghurts are also prone to it because of the lack of gel rigidity [74]. Syneresis was determined mostly using a centrifugation method (≈59% of concerned studies) [13, 25, 26, 33, 40, 47–49, 51, 58] but also using the drainage method (≈41% of studies) ([17, 18, 35, 36, 56, 61, 35, 43]). Using a centrifugation method, different centrifugation speeds and quantity of yoghurt centrifuged were used, making difficult cross‐study comparisons. The centrifugation method has been said to better measure water holding capacity of yoghurt, and the filtration/drainage method provides more realistic results [72]. The drainage method is done either using gravity (opposed to accelerated gravity of centrifugation method) through filtration on a filter paper [17, 18, 43, 56, 61] or by collecting whey after keeping yoghurt in a container at an angle of approximately 45°C [35, 36]. This last approach can produce comparable results in a relatively short time, especially with set yoghurt, and its use is to be encouraged. The drainage method through filtration can better work for stirred yoghurt. Syneresis is generally measured at 45°C–5°C (≈70% of studies) [13, 25, 26, 33, 35, 36, 40, 43, 58, 61], but also at 10°C [51] and 20°C–25°C [47, 49, 56]. Since yoghurt is stored at low temperatures such as 4°C, it is advisable for future studies to measure syneresis at these temperatures in order to reflect the reality.

Regardless of the method which is used, in comparison with reference yoghurts, syneresis decreases when starch is added (Supporting Information, Effect of concentration). The decrease is more important with increasing concentration of starch [18, 48, 54, 56, 58, 83]. Since the increase of the concentration of starch is also associated with an increase of apparent viscosity, it can be said that the observed decreases of syneresis with increasing concentration of starch are due to an increase of viscosity of yoghurt. Therefore, the same reason (increase of total solids content) can justify such observations. However, it is worth noting that syneresis is increased when acid hydrolysed starch is used (in comparison with reference yoghurt made without adding starch) [40].

When comparing starches from different sources at similar concentrations, in comparison with yoghurt prepared without adding starch, it seems that sweet potato starch is more efficient in syneresis reduction than maize starch [35, 36], which in turn is more efficient than cassava starch [49], the latter being more efficient than taro starch [43]. Maize starch is more efficient in syneresis reduction than chickpea bean starch, which is more efficient than Turkish bean starch, potato starch being the less efficient [35, 36]. When considering the modification done on starches, modified starches are generally more efficient in syneresis reduction than native starches [13, 26]. An exception to this seems to exist for potato since its native starch is more efficient in syneresis reduction than its enzymatically modified starches [34].

### 5.4. Effect of the Addition of Starch on Rheological Properties of Yoghurt

Many authors have assessed the effect of the addition of starch on rheological properties of yoghurts [35, 36, 43, 48, 52, 54, 56, 57]. Properties that were assessed are firmness, consistency, resilience, hardness, chewiness, springiness, cohesiveness, adhesiveness, gumminess, storage, and loss modulus (Supporting Information, Rheological parameters). Gel hardness/firmness is the measure of the force needed to deform the gel during compression and is related to its structure and strength [35, 36]. Cohesiveness is defined as the strength of hydrogen bonding of the gel and the degree to which food gels can be deformed before it breaks [35, 36]. Adhesiveness is defined as the work required to overcome the attractive forces between the surface of a food and the surface of other materials with which the food comes into contact [35, 36]. Gel springiness is a textural parameter, which is related to elasticity of a sample [35, 36]. Gumminess (which is the equivalent of chewiness but is used for semisolid food like yoghurt) measures the energy required to masticate [84]. Consistency is somehow similar to firmness. This means that hardness, firmness and consistency can be grouped and considered as measuring the same rheological parameter. In the same trend, chewiness and gumminess can be considered as the same characteristics.

From rheological point of view, yoghurt without added thickener, made from milk with variable total solids content (from 9.3% to 22.7% for example and even beyond) is a non‐Newtonian viscoelastic material, which has a yield stress and a shear thinning/pseudoplastic behavior when sheared and a slow recovery after shearing is stopped [58, 72, 80, 82]. It can be a viscoelastic fluid in the case of stirred and drinking yoghurt or a viscoelastic solid in the case of set yoghurt [72]. It is not a true thixotropic material since structural breakdown due to shear is not completely reversible when shearing is stopped [72]. The addition of starch within the concentration used in numerous studies (0.1%–7%, Table 1) does not change these rheological characteristics of yoghurt [13, 26, 33, 35, 36, 43, 52, 54, 62]. An exception seems to exist when distarch phosphate is added during yoghurt production and in that specific case, a shear thickening behavior is observed [26]. However, starches from different sources have different effects on more precise characteristics of yoghurts.

#### 5.4.1. Effect of the Addition of Starch on Hardness/Firmness/Consistency of Yoghurts

When compared with reference yoghurt prepared without starch, the addition of starch generally increases the firmness, hardness and consistency of yoghurts, and increasing the concentration of starch increases these characteristics (Supporting Information, Rheological parameters) [35, 36, 43, 48, 52, 54]. This is not due to an increase of total solids content since the comparisons were done by some authors using control yoghurts prepared using the same total solids content [35, 36]. However, a decrease of firmness (with gelatinized waxy maize starch) [57] and hardness [35, 36, 52] was also observed. The modification cannot justify this observation since gelatinized cassava starch has been shown to also increase yoghurt firmness [48]. The decrease of hardness can be justified by a concentration effect since it is observed at concentrations of starch beyond 1% (Supporting Information, Rheological parameters).

When comparing the different sources of starch, at the same concentration, cassava starch produces firmer yoghurts than taro starch [43]. At low concentration of starch (like 0.1%), maize starch seems to have the highest effect on hardness, followed by sweet potato starch, chickpea beans starch, Turkish beans starch, and potato starch [35, 36]. At high concentration of starch (1% and beyond), it seems that maize and cassava starches produce the lower decrease of hardness (followed by potato and waxy maize) [52] or potato and sweet potato (followed by maize, Turkish beans, and chickpea beans) [35, 36]. From these comparisons, it seems that maize starch produces harder/firmer/consistent yoghurts than cassava starch, which in turn is more efficient than taro starch.

#### 5.4.2. Effect of the Addition of Starch on the Gumminess/Chewiness of Yoghurts

Gumminess/chewiness of yoghurt decreases [35, 36, 52, 56] or increases [35, 36, 52, 56] with addition of starch, and the variation is more important with increasing concentration of starch (Supporting Information, Rheological parameters). The variation seems to be strongly dependent on the concentration of starch. In this respect, at low concentrations of starch (generally below 0.2%), yoghurt gumminess/chewiness decreases when compared with reference yoghurts made without adding starch [35, 36, 56]. At concentration from 0.2% and beyond, gumminess/chewiness generally increases [52, 54]. The exception observed (decrease of gumminess/chewiness at concentration of starch of 1%) [35, 36] can be explained by the fact that the control yoghurt in that study is made with gum (okra seeds extract); the addition of starch will obviously decrease the gumminess/chewiness of yoghurts.

The source of starch also has an effect on yoghurt gumminess. In this respect, regardless of the concentration, the variation of gumminess (in comparison with reference made without adding starch) is dependent on the day that the measure is done. At low concentration of starch (0.1%), on Day 0, maize starch produces the highest increase of gumminess, followed by potato starch, sweet potato starch, chickpea beans starch, and finally Turkish beans starch [35, 36]. On Day 7, legume starches (chickpea and Turkish beans) produced the highest increase of gumminess, followed by maize, sweet potato and potato starches; these latter three sources allowed for a reduction in yoghurt gumminess/chewiness [35, 36]. On Day 15, chickpea bean starch and sweet potato starch increase yoghurt gumminess/chewiness, whereas potato, maize and Turkish bean starch reduce yoghurt gumminess/chewiness (Supporting Information, Rheological parameters). At higher concentrations of starch (1.5%), on Day 0, waxy maize and maize starches produced the highest increase of gumminess/chewiness, followed by cassava starch and then potato [52]. On Day 7, maize starch produced the highest increase of gumminess/chewiness, followed by potato starch, waxy maize starch, and cassava starch, with the use of all these three starches leading to a reduction of gumminess/chewiness in comparison with the control. On Day 21, when compared with control yoghurts, there is always an increase of chewiness/gumminess, the highest increase being obtained with potato starch, then waxy maize starch, cassava starch, and maize starch [52].

#### 5.4.3. Effect of the Addition of Starch on the Cohesiveness, Adhesiveness, and Springiness of Yoghurts

The effect of the addition of starch on yoghurt cohesiveness seems to be dependent on the concentration of starch used. In this respect, at concentrations of starch below or equal to 1%, the addition of starch generally decreases yoghurt adhesiveness (when compared with reference yoghurt), the decrease being more important with increasing concentration of starch [35, 36, 48, 56]. At concentrations of starch beyond 1%, an increase of yoghurt adhesiveness is observed [52, 54]. An exception seems to exist for that rule since esterified pearl millet starch produces more cohesive yoghurts at all the concentrations used (0.5%–2%). The source of starch also has an influence on yoghurt cohesiveness and this is dependent on the day of analysis and the concentration of starch used [35, 36, 52]. At low concentrations of starch (0.1% for example), the highest reduction of cohesiveness is obtained with Turkish bean starch on Day 0 and with potato on Days 7 and 15 [35, 36]. At higher concentrations of starch (1.5% for example), on Day 0, cassava produces the highest increase of cohesiveness, whereas on Days 7 and 21, waxy maize produces the highest increase of cohesiveness [52].

The effect of the addition of starch on the adhesiveness of yoghurts is following the same trend as the effect on the cohesiveness, and the comments done there remain valid here. The effect of the source of starch on adhesiveness is more homogeneous. In this respect, at starch concentration up to 1%, the reduction of adhesiveness is more important for potato starch, then sweet potato/Turkish beans starch and maize, regardless of the day that the analysis is done [35, 36]. At a concentration of starch of 1.5%, the increase of adhesiveness is more important for waxy maize starch on Day 0, potato starch on Day 7, and cassava starch on Day 21 [52] (Supporting Information, Rheological parameters).

The addition of starch to yoghurt increases the springiness of yoghurt, the increase being more important with increasing concentration of starch [54, 56].

#### 5.4.4. Effect of the Addition of Starch on Rheological Model Parameters of Yoghurts

The different rheological models have been used in order to represent the relationship between shear rate and shear stress of yoghurts with added starch are the Herschel–Bulkley model [26, 54, 62, 83], the power law model [35, 36, 43, 54], and the Casson model [54]. When comparing all these models, some authors [54] showed that the Herschel–Buckley model better fits these rheological data (highest *R* ^2^). This is obvious since this model is associated with a yield stress, a consistency index, and a flow behavior index, all these parameters being the characteristics of yoghurt.

Generally, the addition of starch to yoghurt (when compared with yoghurts made without starch) increases the consistency index and the yield stress while decreasing the flow behavior index (Supporting Information, Rheological parameters) [26, 35, 36, 54, 58, 62, 83]. Increasing the concentration of starch generally increases the consistency index and yield stress while decreasing the flow behavior index [26, 54, 58, 62, 83]. These observations clearly confirm the fact that the addition of starch increases the consistency/firmness/hardness of yoghurts while decreasing their flowing behavior (in comparison with reference yoghurt). This is observed for both stirred and set yoghurts. This effect is not due to an increase in total solids content since some studies were done by comparing samples prepared using the same total solids content [35, 36]. When comparing starches from cassava and taro, it is clear that cassava starch produces more consistency in yoghurts than taro starch [43]. When comparing starches from many sources (potato, sweet potato, maize, chickpea beans, and Turkish beans), maize starch generally produces the highest consistency in yoghurts [35, 36].

These results are confirmed by the fact that for all the studies where the storage modulus (G ^′^) and the loss modulus (G ^″^) are determined, the elastic modulus is always higher than the loss modulus (Tan *δ* = G^″^/G^′^ is always lower than 1), showing that yoghurts with added starches are more elastic than viscous [13, 26, 27, 62]. This is independent of the type of yoghurt since the results are similar for stirred and set yoghurt (Table 2).

### 5.5. Effect of the Addition of Starch on Sensory Properties of Yoghurts

Sensory evaluations of yoghurts done by adding starch were mostly preference tests (≈74% of studies), but also descriptive tests (≈10% of studies) and tests done by attributing specific scores to specific characteristics (≈16% of studies) (Supporting Information, Sensory evaluation). Preference tests done were mostly the nine‐point hedonic scale ([33, 48, 49, 55, 60] [17, 35, 36, 50]), but also the seven‐point hedonic scale [8], the five‐point hedonic scale [3, 25], and preference ranking test [51]. Descriptive tests were done using anchored nonstructured 10‐cm–long scales [58, 83].

Characteristics of yoghurts with added starch which were assessed are odor/smell, color/appearance, viscosity/thickness, oral firmness, syneresis/wheying off, cuttability, cohesiveness (spoon and oral cohesiveness), mouth thinning, mouth coating, smoothness, ropiness, sourness/acidity, texture, creaminess, consistency, aroma, taste, mouthfeel, flavor, sourness, and overall acceptability (Supporting Information, Sensory evaluation). Amongst these characteristics, those which were mostly studied (minimum of five studies) were the color/appearance, texture, creaminess, mouthfeel, taste, flavor, and overall acceptability.

#### 5.5.1. Effect of the Addition of Starch on the Color/Appearance of Yoghurt

The color of yoghurt samples was also evaluated using L a b coordinates by some authors [34, 47, 51]. Due to the higher sensitivity of the equipment, priority is given to human evaluation in order to better reflect the reality. In this respect, when compared with standard yoghurts prepared without adding starch, the addition of starch either improves or depreciates the appearance/color of yoghurts (Supporting Information, Sensory evaluation). A depreciation is observed when maize or waxy maize starches (native or cross‐linked) [3, 48, 56], yam starches (modified or not) [50], and native cassava starch [47, 50] are used. An improvement of the appearance of yoghurts seems to be observed with modified starches, mostly with esterified pearl millet starch [54], gelatinized cassava starch [48], and cross‐linked starches [60]. From these observations, it seems that maize or waxy maize starches (modified or not), yam starches (modified or not), and native cassava starch depreciate the color/appearance of yoghurts.

#### 5.5.2. Effect of the Addition of Starch on the Texture, Mouthfeel and Creaminess of Yoghurts

When compared with yoghurts prepared without adding starch, the addition of starch generally improves the texture of yoghurts (Supporting Information, Sensory evaluation) [35, 36, 47, 49, 54, 60, 61]. Increasing the concentration of starch generally improves the texture of yoghurts up to the concentration of 1.5%–2%[54, 60].

When compared with yoghurts prepared without starch, the addition of starch generally improves the creaminess of yoghurts [35, 58, 83]. Although a decrease can be observed [36], it is generally not significant (less than 5%).

When compared with reference yoghurts prepared without starch, the addition of starch generally improves the mouthfeel of yoghurts [8, 34–36, 48, 54, 58, 83]. Increasing the concentration of starch generally improves mouthfeel [8, 48, 58, 83] up to the concentration of 1.5% [8] beyond which mouthfeel seems to reduce. These observations are independent of the source of starch. Some authors [62] have shown that starch concentrations beyond 2% produce rough set yoghurts. However, some authors observed a reduction in mouthfeel when starch is added [50].

#### 5.5.3. Effect of the Addition of Starch on the Taste and Flavor of Yoghurts

When compared with control made without adding starch, the addition of native starches (yam, cassava, and maize) and enzymatically modified cassava starch generally improves the taste of yoghurts [8, 47, 49]. However, a depreciation of taste is also observed with native maize starch [55] and gelatinized cassava starch [48]. The variation of taste is generally more important with increasing concentration of starch. The optimum concentration of starch for depreciation of taste seems to be 0.5%[3, 48], whereas it is 1.5% for the improvement of taste, especially in the case that native yam starch is used [8]. Even in the case that a depreciation of taste is observed (when compared with reference yoghurt), it seems to be the case until 14 days after the manufacture of yoghurts since on Day 21 after the production of yoghurt, an improvement of the taste is observed (optimal at 3% of starch) [60]. This means that the taste of yoghurts in general improves with storage duration. It has been shown that the increase of the concentration of starch generally increases the production of acetaldehyde (when compared with control and during storage) [7], this molecule being known, along with many others (acetone, acetoin, and diacetyl) to contribute to the taste/flavor of yoghurts [11, 12]. Fourteen days after the production of yoghurt, the production of these flavor compounds in yoghurts can be more important in samples produced by adding starches.

When compared with yoghurts made without starch, the addition of starch generally improves the flavor of yoghurt [8, 34–36, 54, 61]. The improvement seems to be more important with increasing concentration of starch, the improvement reaching an optimal increase at a concentration of starch of 1.5% [8, 54]. However, a depreciation of flavor has also been reported, especially when using maize starch [3] and cross‐linked waxy maize [56]. Neither the source of starch nor the modification done on starches can justify the decrease since the use of cross‐linked starch, maize, and waxy maize starches has been reported to improve the flavor of yoghurts [35, 36, 49, 60]. The milk fat content of yoghurts might justify these observations. In fact, in one of these studies [56], the control yoghurt is made with full‐fat milk, whereas samples with added starch are made with partially skimmed milk. This is a clear indication of the fact that milk fat significantly improves the flavor and taste of yoghurts.

#### 5.5.4. Effect of the Addition of Starch on the Overall Quality of Yoghurts

When compared with reference yoghurts made without starch, the addition of starch in yoghurts either improves or reduces the overall sensory properties of yoghurts, and these effects are independent of the source of starch and the modification done (Supporting Information, Sensory evaluation).

An improvement of the overall quality is done when starches from different sources (yam [≈6.25% of sources], potato [≈12.5% of sources], sweet potato [≈12.5% of sources], maize [≈18.75% of sources], chickpea beans [≈12.5% of sources], Turkish beans [≈12.5% of sources], cassava [≈18.75% of sources], and pearl millet [≈6.25% of sources]) and eventual modifications (native [≈82.35% of modifications], chemically modified [≈11.7% of modifications], and enzymatically modified [≈5.88% of modifications]) are used in yoghurt production [8, 35, 36, 47, 49, 54, 60]. The improvement of the quality is generally higher with increasing concentration of starch [8, 54, 60]. Further improvement seems not to be obtained at concentrations between 1.5% [8] and 3% [60], inclusive.

A reduction of the quality of yoghurts is observed with starches from different sources (yam [≈14.28% of sources], cassava [≈28.6% of sources], maize and waxy maize [≈57.1% of sources]) and eventual modifications (native [≈57.1% of modifications], chemically modified [≈28.6% of modifications], and gelatinized [≈14.3% of modifications]) [3, 8, 48, 50, 55, 56]. When the reduction of the overall quality is observed, it is more important with increasing concentration of starch [48, 56]. The reduction of the overall sensory quality is generally observed when control yoghurts are made using either full‐fat yoghurts (produced by the authors) [56] or bought from market [8] or by comparing yoghurts made using the same total solids content [50]. Based on these results, it can be said that milk fat significantly contributes to the sensory properties of yoghurts and reducing its quantity is accompanied by a reduction of the sensory characteristics of yoghurts.

In fact, in the literature, the use of thickeners in yoghurt (starch included) is done in low‐fat yoghurts in order to obtain the characteristics (if possible) lost when reducing fat. From a research point of view and in order to reflect the reality, the effect of the addition of starch on the quality characteristics of yoghurt can be understood only if the reference yoghurt is made with full‐fat milk, whereas yoghurts with added starch are made with reduced milk fat and the quantity of starch added must be in such a way that control yoghurt and yoghurts made with starches have the same total solids content as done by some authors [13]. Instead of using powdered milk in order to have the same total solids content as done by some authors [27, 35, 36, 40, 50], milk fat must be used. The reality is that the production of yoghurts using milk with the same milk fat content for all the samples (including the reference yoghurt) generally produces an error due to an increase of the total solids content. The production of yoghurt using milk with different fat content and adjusting the total solids content with powder milk does not allow understanding the effect of the replacement of milk fat by starch on the quality of yoghurts. This means that when starch is added to reduced fat milk and the comparison is done with milk with higher fat content, if the same total solids content is not reached, it must be completed with milk fat. For future studies, in order to understand the effect of the substitution of milk fat in yoghurt by starch, the reference yoghurt can be made with full fat milk, whereas the remaining samples are made with reduced fat milk, the quantity of starch added being in such a way that all the samples will have the same total solid content.

## 6. Effects of Storage Duration of Yoghurts With Added Starch on Characteristics of Yoghurts

The effects of storage duration of yoghurts with added starches have been assessed on the chemical composition (ash, proteins, fat, and carbohydrates contents) [61], pH and titratable acidity [7, 13, 16, 16, 18, 25, 47, 48, 56, 60, 61], flavoring compounds (acetaldehyde) [16, 16], apparent viscosity [16, 16, 48, 52, 56, 61], syneresis [13, 18, 35, 36, 48, 56, 61], textural properties (hardness, cohesiveness, adhesiveness, springiness, gumminess, and chewiness) [35, 36, 52, 56], consistency index and flow behavior index [35, 36], microorganisms′ growth [48], and sensory characteristics [16, 16, 18, 60] of yoghurts.

### 6.1. Effect of Storage Duration of Yoghurts With Added Starch on the Chemical Composition

With increasing storage duration (up to Day 10), an increase of ash content (up to 6.67%), protein content (up to 6.52%), fat content (up to 6.67%), and carbohydrate content (up to 1.9%) are observed [61]. At the same time, a slight decrease of moisture content is observed [61]. When compared with yoghurt made without adding starch, the increase of these compounds is reduced by about 50%. These variations probably indicate that the activity of microorganisms continues during storage and that this activity is reduced in yoghurts with added starch.

### 6.2. Effect of Storage Duration of Yoghurts With Added Starch on the pH and Titratable Acidity

The pHs of yoghurts generally decreases during storage (in a refrigerator), the decrease being more important with increasing storage duration and is generally associated to a concomitant increase of titrable acidity (Supporting Information, Effects of time) [7, 13, 16, 16, 25, 47, 48, 51, 55, 56, 60, 61]. The variation (decrease of pH or increase of acidity) does not stop even 28 days after the production of yoghurts [7, 16, 16, 48, 60]. The reduction of pH is less important with increasing concentration of starch [7, 18, 56, 60]. Since pH reductions are due to more important production of lactic acid, this seems to confirm the reduction of lactic bacteria growth with increasing concentration of starch. An exception (more important reduction of pH with increasing concentration of starch) is observed in the case of ensete starch modified using alcoholic alkaline modification [25]. This can be explained by the fact that alkaline modification increases the pH of starch. The differences which are observed with these characteristics for different studies (Supporting Information, Effects of time) are probably due to differences observed in concentrations of lactic bacteria used, high with high concentration/load of microorganisms (3%) [18] and low with low concentration/load of microorganisms (2%) [60] (Table 2).

### 6.3. Effect of Storage Duration of Yoghurts With Added Starch on the Apparent Viscosity and Syneresis of Yoghurts

Storage duration has opposite effects on the apparent viscosity of yoghurts with added starch. In this respect, although some authors observe a decrease of apparent viscosity with increasing storage duration [48, 52, 56, 61], others observe an increase of apparent viscosity with increasing storage duration [33, 52]. The increase of apparent viscosity is observed when yam, potato, maize, and waxy maize starches are used, whereas the decrease is observed when native maize, native, and gelatinized cassava starches are used. These studies seem to clearly show that a decrease of apparent viscosity is always observed when cassava starch is used. The apparent viscosity seems stable (variation of less than 5%), no matter the concentration of starch (0%–3% for the study), when taro starch is used [16, 16]. This decrease is not only due to starch since a reduction of apparent viscosity with increasing storage duration is also observed for yoghurt without starch [48, 56, 61]. The addition of starch reduces the decrease of apparent viscosity with increasing storage duration [48, 61], highest concentrations of starch being associated with lesser reduction of viscosity [48].

Beyond the fact that some sources of starch (yam and taro starches for which an increase of apparent viscosity is observed) are apparently more stable during yoghurt storage, the reduction of apparent viscosity was always observed at concentrations of starch ≤ 1*%* [48, 56, 61]. At higher concentrations (1.5%), an increase of the apparent viscosity seems to be observed regardless of the source of starch [52]. Even at these higher concentrations, the sources of starch seem to have different effects. In this respect, the viscosity seems maximal on Day 7 (after production) for maize and potato; Beyond that time, the viscosity drops to values lower than the first day (potato starch) or is constant (maize starch). When waxy maize is used, beyond 20 days after the production of yoghurt, the apparent viscosity still continues to increase [52]. These results seem to indicate that maize starch is stable during storage of yoghurt when compared with other sources of starch and that cassava starches (native or gelatinized) are the less stable starches. They also indicate that waxy maize is more stable than native maize, meaning that the proportion of amylopectin of starch (100% for waxy maize) can be considered when choosing the source of starch to use.

With respect to syneresis, it varies in opposite directions. Some authors have observed its decrease with increasing storage duration [18, 24, 25, 47, 51, 55, 56]. Increasing the concentration of starch generally reduces the susceptibility of resulting yoghurts to syneresis [25]. Other authors have observed an increase of syneresis with increasing storage duration [13, 35, 36, 48, 61]. The source of starch cannot justify the opposite variations observed since using maize, cassava, and sweet potato starches, an increase and a decrease of syneresis are observed. The type of yoghurt cannot also justify these facts done since an increase and a decrease of syneresis are observed for set yoghurt (Table 2). The observed differences can be explained by the concentration of starches used in the different studies. The increase of syneresis with storage duration (generally associated with a decrease of apparent viscosity) is observed in studies done using starch concentrations, which are lower or equal to 1% (Table 1) [13, 35, 36, 48, 61]. The decrease of syneresis (generally associated with an increase of apparent viscosity) is observed in studies, where the concentrations of starches used were between 1% and 3% (Table 1) [18, 24, 25, 47, 51, 55]. This is consistent with observations done for apparent viscosity and seems to show that increasing the total solids content of milk decreases the susceptibility of yoghurts to syneresis through an increase of viscosity.

### 6.4. Effect of Storage Duration of Yoghurts With Added Starch on Textural Properties of Yoghurts

Hardness, cohesiveness, and gumminess/chewiness increase with increasing storage duration of yoghurt, and this is observed whether starch is added to yoghurts or not (Supporting Information, Effects of time) [35, 36, 52, 56]. The increase of these parameters is generally more important with increasing concentration of starch. Yoghurt (with added starch) cohesiveness seems maximum 7 days after yoghurt production [35, 36, 52, 56]. Springiness decreases with increasing storage duration, whereas the variation of adhesiveness seems to be dependent on the concentration of starch [35, 36, 52, 56]. At a concentration of starch of 1%, adhesiveness decreases with increasing storage duration [35, 36], whereas at higher concentrations, it increases with increasing storage duration [52]. Starches from different sources have different intensities of effects [35, 36, 52].

### 6.5. Effect of Storage Duration of Yoghurts With Added Starch on Sensory Characteristics

Sensory characteristics of yoghurts with added starches have been assessed on different days by many authors. Evaluation were done on the appearance, syneresis, smoothness, firmness, mouthfeel, smell, texture, flavor, aroma, acidity, and taste of yoghurts [16, 16, 18, 48, 51, 56, 60].

For yoghurt made without starch, some authors showed that some sensory characteristics (aroma and taste) remain the same with increasing storage duration [16, 16], whereas others show that there is an improvement in many sensory characteristics (appearance, body and texture, flavor, and acidity) [16, 16, 18].

When starch is added to yoghurts, the effect on sensory properties seems to be dependent on the concentration of starch and the source of starch. In this respect, with concentrations of starch (yam, cassava, maize, and waxy maize starches) ≤ 1*%*, there is not a significant change (change of less than 5%) of sensory properties (color/appearance, firmness, flavor, smoothness, taste, mouthfeel, and overall acceptability) of yoghurts with increasing storage duration [18, 48, 51, 56]. When sweet potato starch is used, there is an improvement of color/appearance, body and texture, and acidity with increasing storage duration, and this is observed up to the concentration of starch of 0.5% or 0.75%–1% (for acidity), beyond which these sensory characteristics are maximal and constant with increasing storage duration [18]. When taro starch is used, at higher concentrations, there is no change of aroma (2.5%–3%), taste (2%–3%), and flavor (1.5%–2%) with increasing storage duration [16, 16]. When changes are observed, it is always beyond 14 days and is either a depreciation (concentration of 1.5–2% for aroma, 0.5% and 3% for flavor, and 1.5% for the taste) or improvement (concentration of 1% for aroma, 1% and 2.5% for the taste) of sensory characteristics with increasing storage duration [16, 16].

### 6.6. Effect of Storage Duration of Yoghurts With Added Starch on Lactic Bacteria Growth

The microbial load of yoghurts generally increases with increasing storage duration [16, 16, 18, 25, 55]. When compared with yoghurts made without starch, the addition of starch delays the microbial growth of yoghurt, the delay being more important with increasing concentration of starch [18, 48]. When comparing starches from different sources, at same concentration more important delay is obtained with gelatinized cassava starch than with native maize starch [48]. This might justify higher pHs and lower acidity observed when starch is added to yoghurt (Section 5.2). The increase of lactic bacteria seems to be due to *L. delbrueckii* subsp. *bulgaricus* only since their increase was observed during storage, whereas a decrease is observed for *S. thermophilus* [51]. During yoghurt production, *S*. *thermophilus* grow faster in the early incubation period, producing carbon dioxide and formic acid, which stimulate the growth of *L. delbrueckii* subsp. *bulgaricus*, resulting into a production of more lactic acid, which further decreases the pH up to values around 4 [11]. This low pH, which is not favorable to *S. thermophilus* and which decreases with increasing storage duration, will certainly decrease the growth of *S. thermophilus*. There is a variety effect since the load of *L. delbrueckii* subsp. *bulgaricus* decreases with increasing storage duration for starch obtained from one variety of yam, whereas a decrease is observed for starch obtained from another variety [51]. The general increase of microbial load during storage justifies why the increase of acetaldehyde content of yoghurts during storage is observed, although its production seems maximal 14 days after yoghurt is produced [7].

## 7. Conclusion

Starches from maize seeds and cassava roots, native or chemically modified, are the most used in studies involving starches and yoghurt. When compared with reference yoghurt made without adding starch, the addition of starch increases the carbohydrates content of yoghurts while decreasing the fat content, protein content, and mineral content of yoghurts. This addition of starch generally leads to an increase of the pH and apparent viscosity of yoghurts. The effects of the addition of starch on the rheological properties of yoghurt depend on the concentration of starch used. Fat reduction in yoghurts is associated with a reduction of sensory characteristics (taste, flavor, and overall quality) of yoghurts. The addition of starch depreciates the color/appearance of yoghurt (maize, yam, and cassava starches); improves or depreciates the flavor, taste, and overall quality of yoghurt (depending on the concentration used); and improves yoghurt mouthfeel, texture, and creaminess. The addition of starch delays the increase of the nutrient content of yoghurt during storage as well as the pH reduction of yoghurts. At lower concentrations of starch (lower or equal to 1%), the apparent viscosity of yoghurts decreases with increasing storage duration (while at the same time, syneresis increases with increasing storage duration). At higher concentrations of starch (greater than 1%), the reverse is observed. Yoghurt hardness, cohesiveness, and gumminess/chewiness increase with increasing storage duration. With respect to sensory characteristics, at lower concentrations of starch (lower or equal to 1%, 0% included), there is no significant change in sensory properties of yoghurts during storage and at higher concentrations of starch; sensory characteristics are maximal and stable with increasing storage duration.

## Funding

No funding was received for this manuscript.

## Conflicts of Interest

The authors declare no conflicts of interest.

## Supporting information


**Supporting Information** Additional supporting information can be found online in the Supporting Information section. Supporting information of this work provides the computed data obtained from the different articles that were used in order to determine the effects of the addition of starches. Data for the effects of the addition of starches on rheological (2) and sensory properties (1) of yoghurt are found on separate spreadsheets (Rheological parameters, Rheological model, and Sensory evaluation). Data of the chemical composition and physicochemical properties are combined in one spreadsheet (Effect of concentration). Data of the effect of storage duration on characteristics of yoghurt are found in another spreadsheet (Effects of time) or each aspect is found in a separate spreadsheet. These data are also organized depending on the source of starch (Effect Conc. and source and effect time and source spreadsheets), the modification done on starches (Effect Conc. and modification and Effect time and modification spreadsheets), the day of analyses (Effects Conc. and day), and the day of analysis and concentration of starch used (Effect time and concentration).

## Data Availability

The data that support the findings of this study are openly available in figshare at https://figshare.com/account/articles/30371647?file=58784353 (Reference Number 58784353).
